# VEGF111b, a C-terminal splice variant of VEGF-A and induced by mitomycin C, inhibits ovarian cancer growth

**DOI:** 10.1186/s12967-015-0522-0

**Published:** 2015-05-20

**Authors:** Xiuli Li, Fang Gu, Chenguang Niu, Yuanfen Wang, Zhongyu Liu, Na Li, Bing Pan, Dan He, Jian Kong, Shaobo Zhang, Xu Wang, Yuanqing Yao, Lemin Zheng

**Affiliations:** Department of Obstetrics and Gynecology, Chinese PLA General Hospital, Beijing, China; Department of Obstetrics and Gynecology, Beijing Chaoyang Hospital, Beijing, China; The Institute of Cardiovascular Sciences and Institute of Systems Biomedicine, School of Basic Medical Sciences, and Key Laboratory of Molecular Cardiovascular Sciences of Ministry of Education, Peking University Health Science Center, Beijing, 100191 China; Department of Obstetrics and Gynecology, Affiliated Hospital of Academy of Military Medical Sciences, Beijing, 100071 China; Department of Hepatobiliary Surgery, Beijing Chaoyang Hospital, Capital Medical University, Beijing, 100043 China; Department of General Surgery, PLA 180th hospital, Fujian, China

**Keywords:** VEGF111b, Ovarian cancer, Anti-tumor, Cell cycle, VEGF-R2

## Abstract

**Background:**

Alternative splicing of VEGF-A gives rise to two families – the pro-angiogenic VEGFxxx family and the anti-angiogenic VEGFxxxb family that differ by only six amino acids at their C-terminal end. The first verified and widely reported VEGFxxxb family member is VEGF165b, and here VEGF165b is a positive control.

**Methords:**

VEGF111b mRNA was detected in ovarian cancer cell lines SKOV3 and OVCAR3 by RT-PCR. Western blot was used to detect VEGF111b and VEGF165b protein in the CMs and lysates of OVCAR3 cells. MTT and colony formation assay were used to detect the short-term and long-term proliferation inhibition ability of ovarian cancer cells with VEGF111b overexpression. Cell-cycle analysis was performed to further characterize VEGF111b inhibition effects. VEGF111b signaling on ovarian cancer cells were determined by western blot. The expression levels of Ki67, PCNA, CD31 and VEGF in VEGF111b overexpression xenograft model were detected by immunohistochemistry.

**Results:**

Under the effect of mitomycin C, we identify a new member of VEGFxxxb family-VEGF111b in ovarian cancer cell lines. SKOV3 and OVCAR cells were transfected with empty lentivirus, VEGF111b or VEGF165b lentivirus. VEGF111b and VEGF165b overexpression inhibits proliferation of the ovarian cancer cells, but inhibition effect of VEGF111b is slightly less efficient than VEGF165b. Cell cycle analysis was further used to elucidate the mechanism involved in the inhibition effect. Further, we detected the expression of VEGF-R2 in SKOV3 and OVCAR3 cells, and shown that VEGF111b might bind to conventional VEGF-R2 with the results of reducing VEGF-R2 tyrosine phosphorylation and downstream signaling to have anti-tumor effects. *In vivo* VEGF111b overexpression inhibits ovarian cancer growth in xenograft mice.

**Conclusion:**

Our results show that VEGF111b, as a new member of VEGFxxxb family, with similar properties to VEGF165b, plays potent anti-tumor effect *in vitro* and *in vivo* that can target the VEGF-R2 and its signaling pathway to inhibit ovarian tumor growth. This also opens a new avenue for treating ovarian cancer.

**Electronic supplementary material:**

The online version of this article (doi:10.1186/s12967-015-0522-0) contains supplementary material, which is available to authorized users.

## Background

Ovarian cancer accounting for third in gynecological tumors presents at advanced stage in around 75 % of women and is the fifth leading cause of gynecological cancer-related deaths [1]. Despite improvements in treatments such as chemotherapy, the 5-year survival rate from the disease in women was only 36 % [2]. The high mortality of ovarian cancer urgently expected much effective interventions. In recent years, molecular therapies shows promise for ovarian cancer treatment in preclinical and clinical setting. Among these, anti-angiogenic therapy has attracted much more attention [3]. The efficacy of anti-angiogenic therapy was confirmed in four randomized, double-blind phase III trials, both as front-line treatment (GOG-0218 and ICON7) and in patients with recurrent ovarian cancer (OCEANS and AURELIA). In the four studies, meaningful improvement in PFS was achieved with the addition of bevacizumab to standard chemotherapy in ovarian cancer treatment [4].

Vascular endothelial growth factor (VEGF-A) has been identified in a number of studies as a key component in tumor growth and angiogenesis [5]. It has been proved that, the positive expression of VEGF and its receptor rate in malignant ovarian cancer tissues was significantly higher than that in benign ovarian tumor tissues [6]. Structure and alternative splicing of the VEGF-A gene were shown [7]. Following the initial identification of VEGF-A [8], many splice isoforms were subsequently identified, eventually forming two known families of protein isoforms – the pro-angiogenic VEGFxxx family and the anti-angiogenic VEGFxxxb family [9, 10], where xxx denotes the number of amino acids of the secreted isoform, such as VEGF-121, VEGF-165 (the dominant pro-angiogenic isoform), VEGF-165b, and others [11]. VEGF-A is generated as multiple isoforms by alternative splicing of mRNA from 8 exons which gives rise to two families of isoforms, the VEGFxxx and VEGFxxxb isoforms that differ by only six amino acids at their C-terminal end [12]. VEGFAxxxb isoforms are formed by distal splice site (DSS) in exon 8, forming an mRNA containing 18 bases coded by exon 8b in place of the 18 bases of exon 8a of VEGFxxx isoforms [13]. Exons 8a and 8b both code for six amino acids, exon 8a for CDKPRR and exon 8b for SLTRKD. The VEGFxxx isoforms are demonstrated to be pro-angiogenic and the VEGFxxxb isoforms are anti-angiogenic [13]. This anti-angiogenic activity is regulated by receptor binding, but only weak receptor activation and inhibition of downstream VEGF-R2 signaling [14, 15].

VEGF111was reported as a new VEGFxxx family member in 2007, and demonstrated to be induced in the condition of mitomycin C [16]. In our published study, we first discover VEGF111b protein under the induction of mitomycin C, and confirm its anti-angiogenic effect *in vitro* [7]. To determine whether VEGF111b inhibit ovarian cancer cell growth *in vitro* and whether VEGF111b exerts inhibitory effects on tumor growth in xenograft mice, we present descriptive expression data and functional data on cell proliferation, cell cycle and *in vivo* tumor growth and angiogenesis. We also probed potential mechanism of the inhibitory effect of VEGF111b.

## Materials and methods

### Reagents and antibodies

Mitomycin C was obtained from Sigma-Aldrich (Saint Quentin Fallavier, France). VEGF-R2 pAb (BA0486, 1:250) was purchased from Beyotime (Jiangsu, China). PCNA mAb (PC10, 1:100), Ki67 mAb (7B11, 1:100), VEGF pAb (ZA-0580, 1:100) and CD31 mAb (1A10, 1:75) were purchased from ZSGB-BIO (Beijing, China). The VEGF111b polyclonal antibody (1:100) is our own preparation in a previous study [7]. 165b mAb (MRVL56/1, 1:1000), p44/42 MAPK pAb (3A7, ERK1/2) (1:1000), PI3K mAb (D32A5, 1:1000) and Akt mAb (40D4, 1:1000) were purchased from Abcam (Cambridge, MA, USA). Phospho-p44/42 MAPK (ERK1/2) mAb (Thr202/Thr204,1:1000), phospho - PI3K pAb (Tyr458/Tyr199, 1:1000) and phospho-Akt mAb (Thr 308, 1:1000) were purchased from Cell Signaling Technology (Danvers, CO, USA). Horseradish peroxidase (HRP)-labeled anti-mouse and anti-rabbit secondary antibodies were from Santa Cruz (Dallas, TX, USA).

### Ovary cancer cell lines and groups

Human ovarian cancer cell lines, SKOV3 and OVCAR3 were obtained from the Chinese Academy of Medical Sciences. SKOV3 was cultured in Roswell Park Memorial Institute −1640 culture (RPMI-1640, HyClone). OVCAR3 was maintained in high-glucose Dulbecco’s modified Eagle medium (DMEM, HyClone) with 10 % fetal bovine serum (FBS), 100 U/ml penicillin and 100 μg/ml streptomycin (Life Technologies, Cergy Pontoise, France). The 293 T cells was also obtained from the Chinese Academy of Medical Sciences and maintained in high-glucose Dulbecco’s modified Eagle medium (DMEM, HyClone) with 10 % fetal bovine serum (FBS). Cells were cultured in a humidified atmosphere of 5 % CO_2_ at 37 °C. The human ovarian cancer cells were divided into four groups: (1) control group: without any treatment; (2) empty vector group: the cells were transfected with empty lentivirus vector carrying GFP gene; (3) VEGF111b group; and (4) VEGF165b group: the cells were transfected with full-length VEGF111b or VEGF165b (generated by RT-PCR from SKOV3 cells or OVCAR cells) using lentivirus, each at a dose of 20 MOI and allowed to grow for 48 h. After infection with lentivirus vector carrying GFP gene for 48 h, the expression rate of GFP green fluorescence in the ovarian cancer cell pools all reached 95 %.

### RNA extraction and RT-PCR analysis

SKOV3 and OVCAR3 cells were respectively treated with 100 μg/ml mitomycin C for 24 h, then total RNA was extracted using Trizol reagent (Invitrogen, USA). Complementary DNA was made using oligo dT primer (TransGen, Beijing, China) under the manual of the manufacturer. The cDNA of SKOV3 and OVCAR3 cells was the template, and PCR was performed with initial denaturation at 94 °C for 5 min, followed by 30 cycles of amplification (30 s at 94 °C, 30 s at 55 °C, 1 min at 72 °C), and final extension at 72 °C for 10 min. According to alternative splicing of VEGFxxx and VEGFxxxb families, the VEGF111b mRNA is composed of exons 1–4 and 8b. We designed forward primer of VEGF111b in exon 4, and reverse primer in the junction of exon 4 and 8b. Primers sequences are listed as follows: VEGF111b 5′-CCACTGAGGAGTCCAACATCA-3′(forward); 5′- AATGCAGATGTGACAAGCCGAG −3′(reverse). VEGF165b 5′-GAGATGAGCTTCCTACAGCAC-3′(forward); 5′- TTAAGCTTTCAGTCTTTCCTGGTGAGAGATCTGCA-3′(reverse). GAPDH 5′- CGGAGTCAACGGATTTGGTCGTAT-3′(forward); 5′- AGCCTTCTCCATGGTGGTGAAGAC-3′(reverse). Primers were selected using the NCBI/primer-blast program (http://www.ncbi.nlm.nih.gov/tools/primer-blast/) and were synthesized by Sangon Biotech (Sangon Biotech Co., Ltd, Shanghai, China). PCR products were separated and visualised using 4 % agarose/ethidium bromide gel.

### Lentivirus preparations

The pCDNA 3.1 (+)-VEGF111b plasmid (bought from SinoGenomax) was the template, and PCR was performed with initial denaturation at 94 °C for 5 min, followed by 30 cycles of amplification (30 s at 94 °C, 30 s at 55 °C, 1 min at 72 °C), and final extension at 72 °C for 10 min. Primers for VEGF111b are listed as follows:

- Forward-BamHI: atcggatccgccgccaccATGAACTTTCTGCTGTCTTG
- Reverse-AgeI: atcaccggtggGTCTTTCCTGGTGAGAG

PCR products were separated and visualized using 2 % agarose/ethidium bromide gel. By double digestion with BamHI/AgeI restriction endonuclease, ligation and transformation, we prepared the recombinant lentiviral particles pLV-VEGF111b-EGFP. 293 T cells were plated into 10 cm cell culture dishes and the cell density reached 70 ~ 80 % after 24 h. For each plate in a transfection, dilute 10 μg of DNA (5 μg pLV-VEGF111b-EGFP, 3 μg pHelper 1.0 (Gag/pol/rev plasmid) and 2 μg pHelper2.0 (VSVG envelope plasmid)) into 750 μl serum free, antibiotic free medium. Mix gently. For each plate in a transfection, dilute 30 μl of LipofectamineTM2000 (Invitrogen) into 750 μl serum free, antibiotic free medium and mix. Then, combine diluted DNA and diluted LipofectamineTM 2000 reagent, and incubate at room temperature for 15 min to allow DNA-liposome complexes to form. While complexes are forming, replace the medium on the cells with 5 ml of serum free, antibiotic free medium. Overlay the diluted complex solution onto the rinsed cells. Incubate the cells with the complexes for 5 h at 37 °C in a CO2 incubator. Then, remove the transfection mixture and replace it with complete growth medium. Lentiviral supernatant was collected after 48 h. A second collection was made after a further 24 h. The conditioned medium from the two harvests was combined and cleared by centrifugation at 1500 rpm for 5 min at 4 °C, and then passed through a 0.45 μm pore PVDF Millex-HV filter (Millipore). Concentration of lentivirus using ultracentrifugation was performed with a Beckman optima TM L-90 K centrifuge using a 50.2 rotor. Filtered lentiviral supernatant was added to 36 ml pollyallomer conical tubes (Beckman). Centrifugation was performed for 3 h at 50000 g unless otherwise stated. Supernatant was completely removed and virus pellets were suspended in 200 μl PBS overnight at 4 °C and stored at −80 °C until use.

### Western blot

SKOV3 and OVCAR3 cells were respectively seeded into 6-well plates. Following day, the cells were treated with 100 μg/ml mitomycin C for 48 h, and the remaining cells were respectively transfected as the above and allowed to grow for 48 h. In the VEGF111b polyclonal antibody group, the cells respectively transfected were treated with VEGF111b polyclonal antibody (1:100) and grew for 1 h. Then the cells were lysed using cell lysis buffer (150 mM NaCl, 50 mM Tris–HCl, pH 8.0, 0.1 % SDS, 1 % Triton X-100). The conditioned mediums (CMs) were collected. Equivalent amounts of cell lysates and concentrated CMs were separated by SDS-PAGE gel and transferred onto nitrocellulose membranes. The membranes were blocked in 5 % skimmed milk for 2 h and then incubated with respective primary antibody over night at 4 °C followed by the incubation of the appropriate HRP-conjugated secondary antibody for 2 h at room temperature. The signal was detected with SuperSignal West Pico substrate (Thermo scientific, Rockford, IL, USA).

### Cell viability assay

Cell viability assay was evaluated by 3-[4, 5-dimethylthiazol-2-yl]-2, 5-diphenyl tetrazolium bromide (MTT) assay. The ovarian cancer cells were transfected according to the above grouping and allowed to grow for 48 h. The cells (4 × 10^3^/well) were seeded into 96-well plates. After incubation for 48 h and 72 h, the cells were stained with 20 μl of 5 mg/ml MTT (Ameresco, USA) and lysed in 100 μl DMSO each well. After mixing for 10 min at room temperature, formazan production was determined by measurement of the optical density (OD) at 570 nm using an ELISA plate reader (model 550, BioRad, USA). The average values were determined from quadruplicate readings, and the experiment was repeated in triplicate.

### BrdU proliferation assay

The cell BrdU proliferation assay was conducted according to the manufacturers protocol. After transfection, the ovarian cancer cells (1000/well) were plated in 96-well plates and cultured for 48 h. Subsequently, the cells were labeled with BrdU lateling solution (20 μl/well). After incubation with 200 μl/well FixDenat, the cells were incubated with BrdU-POD working solution for 1.5 h. Then the cells were washed with PBS and substrate solution (TMB) was added. The absorbance of each well was read at 570 nm using an ELISA plate reader (model 550, BioRad, USA). The average values were determined from quadruplicate readings, and the experiment was repeated in triplicate.

### Colony formation assay

Briefly, the cells (1000/well) were seeded into 6-well plates and allowed to grow in complete medium for 2 weeks. The colonies obtained were washed with PBS and fixed in 4 % paraformaldehyde for 20 min at room temperature and then washed with PBS followed by staining with crystal violet. The colonies were counted and compared in four groups.

### Cell cycle analysis by flow cytometry

After transfection, the ovarian cancer cells were plated in 6-well plates at 2 × 10^5^/well. Following the designated treatments, cells were harvested by trypsinization and washed with PBS and fixed in ice-cold 75 % ethanol overnight at −20 °C. Fixed cells were washed, and dissolved in RNAse and subsequently incubated at 37 °C for 30 min. Next, cells were stained with propidium iodide (PI) for 30 min. The DNA content of the cells (1 × 104 cells per experimental group) was determined using a BD Accuri C6 flow cytometer (BD biosciences).

### *In vivo* xenograft models

Four-weeks-old Male BALB/c nu/nu mice were obtained form Vital River Laboratories (Beijing, China) and bred in specific pathogen-free conditions. The animals were housed in an area with a constant humidity of 60 %-70 % and a room temperature of 18 °C-20 °C. All animal experimental procedures were carried out in accordance with protocols approved by Chinese PLA Medical University (Beijing, China). The 10 mice were divided into two groups: empty vector group and VEGF111b group, and each group had 5 mice. The cells were transfected with full-length VEGF111b using lentivirus and empty lentivirus vector. Xenograft tumors were established by subcutaneous injection of 10^6^ cells suspended in 200 μl PBS into the upper right flank of each mouse. Tumor volume, based on caliper measurements, was calculated every 4 days for 20 days according to the ellipsoid volume formula, tumor volume = (the shortest diameter) ^2^ × the largest diameter × 0.525. At the end of the experiments, mice were euthanized, blood samples were collected via cardiac puncture, and tumor tissues were removed for fixation in the 4 % paraformaldehyde for histological examination and immunohistochemical staining.

### Immunohistochemical staining

Tissues were fixed in 4 % paraformaldehyde and subsequently embedded in paraffin. Paraffin-embedded tissue sections were cut into standard 6 μm sections, deparaffinaged in xylene and rehydrated through graded alcohol solutions. Antigen retrieval was performed 10 min at 92 °C in EDTA (10 mmol/l, pH 8.0) in a water bath. Endogenous peroxidases were inactivated by immersing the sections in 0.3 % hydrogen peroxide for 12 min. The sections were blocked with 5 % goat serum for 60 min at 37 °C. The slides were incubated with primary antibodies for overnight at 4 °C. Next, the slides were treated with appropriate HRP-conjugated secondary antibody for 40 min at 37 °C and then developed with 3,3′-diaminobenzidine. Finally, the slides were counterstained with hematoxylin and mounted. The slides were examined with Nikon Eclipse Ti microscope under a 200× objective.

### Statistical analysis

All values were expressed as the mean ± SEM. The data were analyzed using Student’s *t* test, non-parametric Student’s *t* test, or Two -way ANOVA test. P value of <0.05 was considered statistically significant. GraphPad Prism (GraphPad Software Inc., San Diego, California, USA) was used for these analyses.

## Results

### VEGF111b can be induced by mitomycin C in ovarian cancer cells

**Fig. 1:**
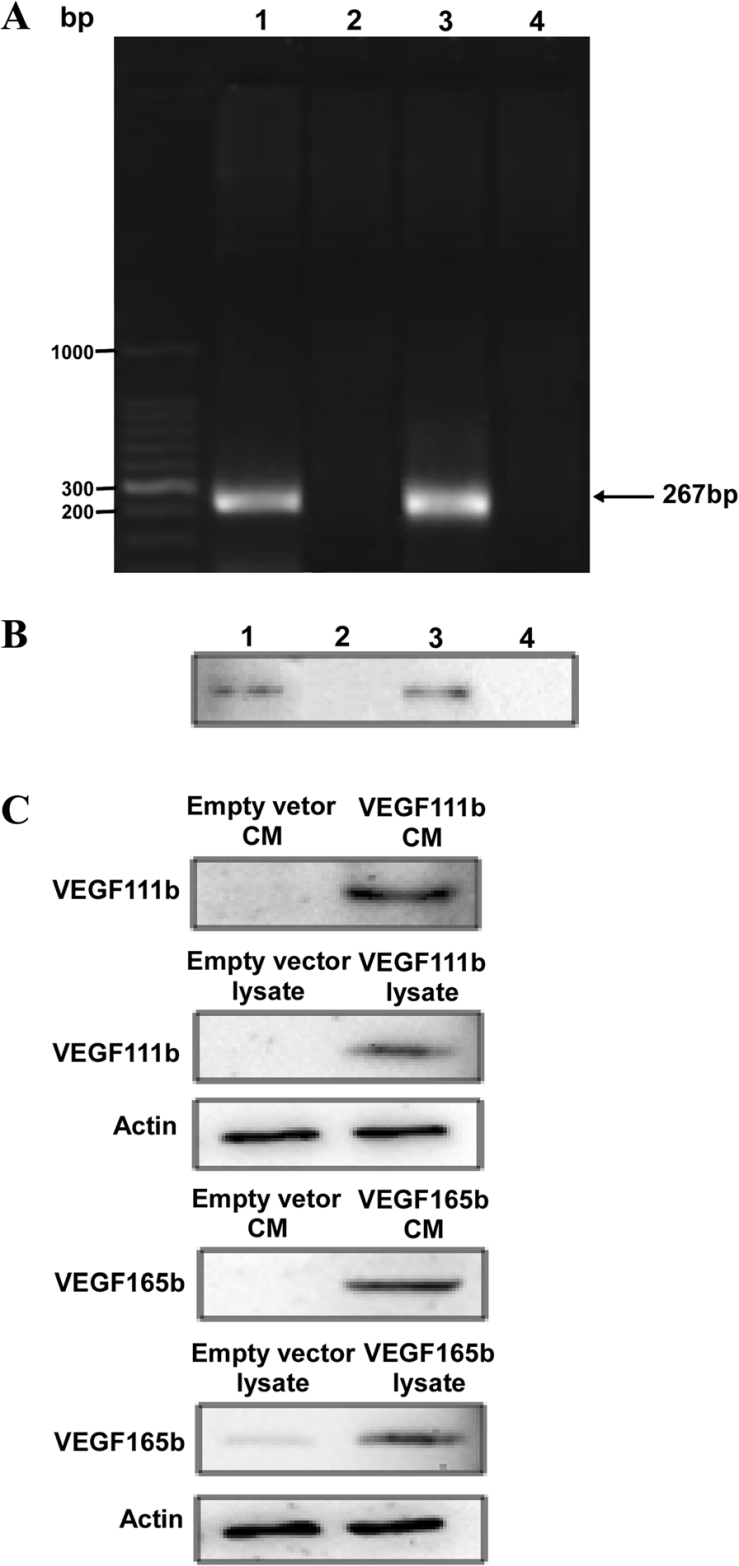
The mRNA and protein expression of VEGF111b could be induced by Mitomycin C in ovarian cancer cells. (**a**) RT-PCR to detect VEGF111b mRNA. After treating with 100 μg/ml mitomycin C, VEGF111b mRNA was observed in ovarian cancer cells SKOV3 (line 1) and OVCAR3 (line 3). But VEGF111b mRNA was not observed in the cells (line 2 and line 4). (**b**) VEGF111b protein was detected in SKOV3 cells (B, line 1) and OVCAR3 cells (line 3) after treating with 100 μg/ml mitomycin C by using VEGF111b polyclonal antibody, but not observed in mitomycin C-unexposed SKOV3 and OVCAR3 cells (line 2 and line 4). (**c**) In VEGF111b and VEGF165b group, VEGF111b and VEGF165b protein were observed in the conditioned mediums and lysates in the OVCAR3 cells transfected with VEGF111b or VEGF165b using lentivirus

We have previously shown that VEGF111b expression is induced in SKOV3 cells after 100 μg/ml mitomycin C treatment, and VEGF111b protein was detected by our homemade VEGF111b polyclonal antibody [7]. And, our homemade VEGF111b polyclonal antibody was validated in Additional file: 1 Figure S1. After mitomycin C treatment, we observed the VEGF111b mRNA product in SKOV3 cells (Fig. 1a, line 1), and in another ovarian cancer cell line OVCAR3 cells (Fig. 1a, line 3). VEGF111b protein was also detected in both SKOV3 cells (Fig. 1b, line 1) and OVCAR3 cells (Fig. 1b, line 3) after mitomycin C treatment. In contrast, there was no expression of VEGF111b protein in mitomycin C unexposed cells (Fig. 1b, line 2 and line 4). After the lentivirus infection with empty vector, VEGF111b and VEGF165b respectively, the CMs and lysates of OVCAR3 cells were collected to determine whether VEGF111b and VEGF165b can be secreted. Consistent with our previous study [7], western blot showed that VEGF111b and VEGF165b protein was present in both CMs and lysates of OVCAR3 cells (Fig. 1c). In contrast, in the cells transfected with empty lentivirus vector, VEGF111b protein was not detected in the medium or lysates.

### VEGF111b overexpression inhibits viability and proliferation of ovarian cancer cells *in vitro*

**Fig. 2:**
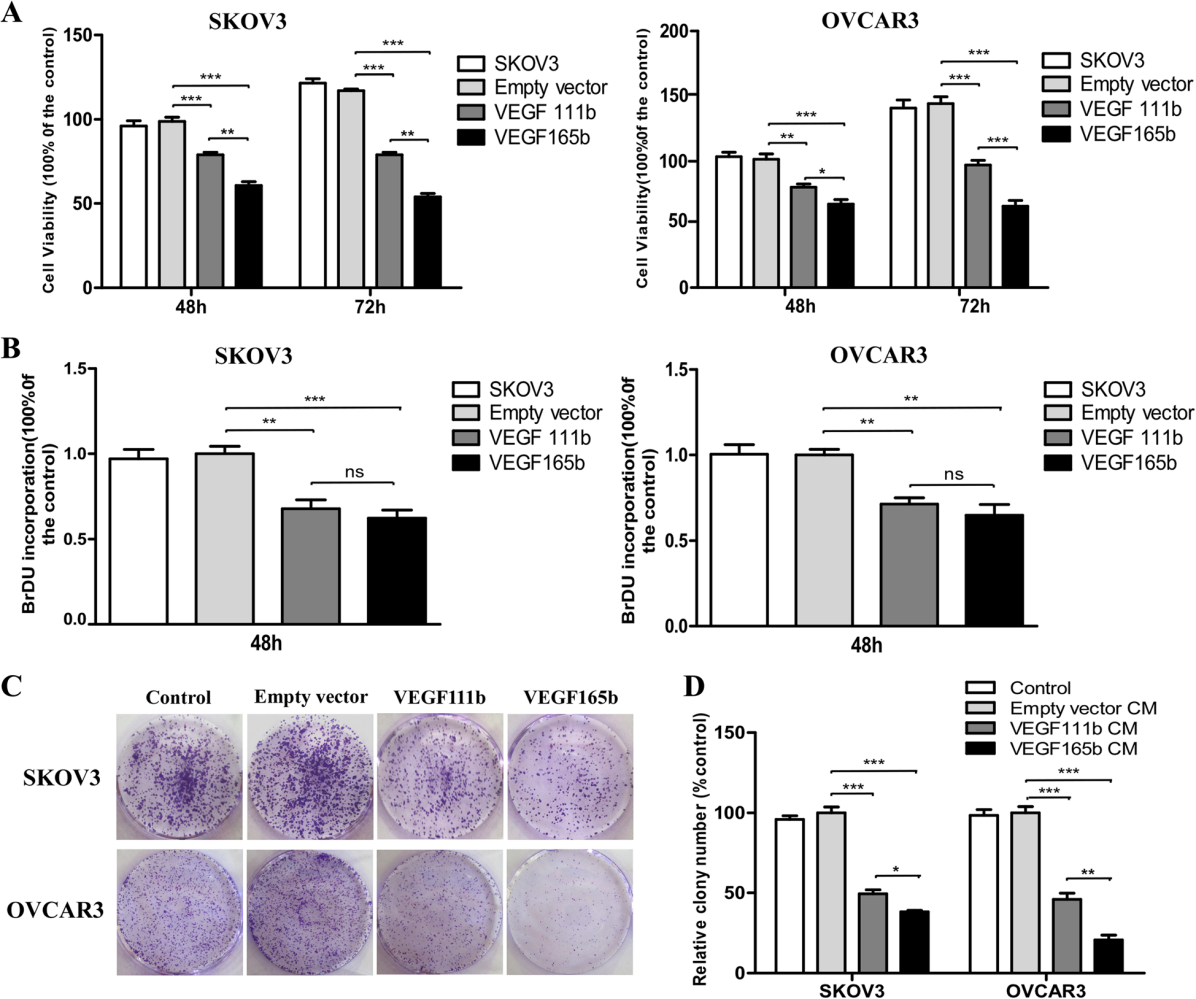
VEGF111b overexpression inhibits proliferation of ovarian cancer cells *in vitro*. (**a**) SKOV3 and OVCAR3 cells were transfected with empty lentivirus and VEGF111b、VEGF165b lentivirus, respectively, and incubated for 48 h and 72 h, cell viability was measured by MTT assay and expressed as percentage in comparison with empty vector (*p < 0.05; ** *p* < 0.01; ***p < 0.001). (**b**) SKOV3 and OVCAR3 cells were transfected with empty lentivirus and VEGF111b、VEGF165b lentivirus, respectively, and incubated for 48 h, cell proliferation was measured by BrdU assay and expressed as percentage in comparison with empty vector (**p < 0.01; ***p < 0.001; ns, no significance). (**c-d**) SKOV3 and OVCAR3 cells were transfected and allowed to grow in complete medium for 2 weeks. The colonies were assessed. VEGF111b inhibited the colony formation of SKOV3 and OVCAR3 cells in comparison with empty vector (*p < 0.05; ** *p* < 0.01; ***p < 0.001). Error bars represent the SEM of data obtained in three independent experiments

To determine the inhibition effect of VEGF111b on ovarian cancer cells viability and proliferation, SKOV3 and OVCAR3 cells were incubated for 48 h and 72 h after the infection of empty vector, VEGF111b and VEGF165b lentivirus. Then MTT and BrdU assay were performed. In MTT assay, VEGF111b inhibited viability of SKOV3 cells with a 19 ± 4 %, and 38 ± 6 % decrease at 48 h and 72 h, respectively (vs empty vector, *P* < 0.001; Fig. 2a), and OVCAR3 cells with a 21 ± 4, and 37 ± 7 % decrease respectively (vs empty vector, *P* < 0.01, *P* < 0.001; Fig. 2a). Moreover, VEGF165b, as a positive control, inhibited the cell viability more than that of VEGF111b (*P* < 0.05, *P* < 0.01, *P* < 0.001; Fig. 2a). In BrdU proliferation assay, VEGF111b inhibited the proliferation of SKOV3 cells with a 32 ± 9 % decrease at 48 h (vs empty vector, *P* < 0.01; Fig. 2b), and OVCAR3 cells with a 29 ± 6 % decrease at 48 h respectively (vs empty vector, *P* < 0.01, *P* < 0.01; Fig. 2b). However, the inhibition effect of VEGF111b and VEGF165b on SKOV3 and OVCAR3cells proliferation had no significant difference. To determine the long-term inhibition effect of VEGF111b overexpression, cells were allowed to grow in complete medium for 2 weeks. There was lower number of colonies in VEGF111b group than empty vector group (Fig. 2c). VEGF111b inhibited the colony formation of SKOV3 and OVCAR3 cells (vs empty vector, *P* < 0.001; Fig. 2d). Significant results were also found in VEGF165b group (vs empty vector, *P* < 0.001; Fig. 2d) and the inhibition level of VEGF165b was more than that of VEGF111b (*P* < 0.05, *P* < 0.01; Fig. 2d).

### VEGF111b overexpression induces cell cycle arrest of ovarian cancer cells

**Fig. 3:**
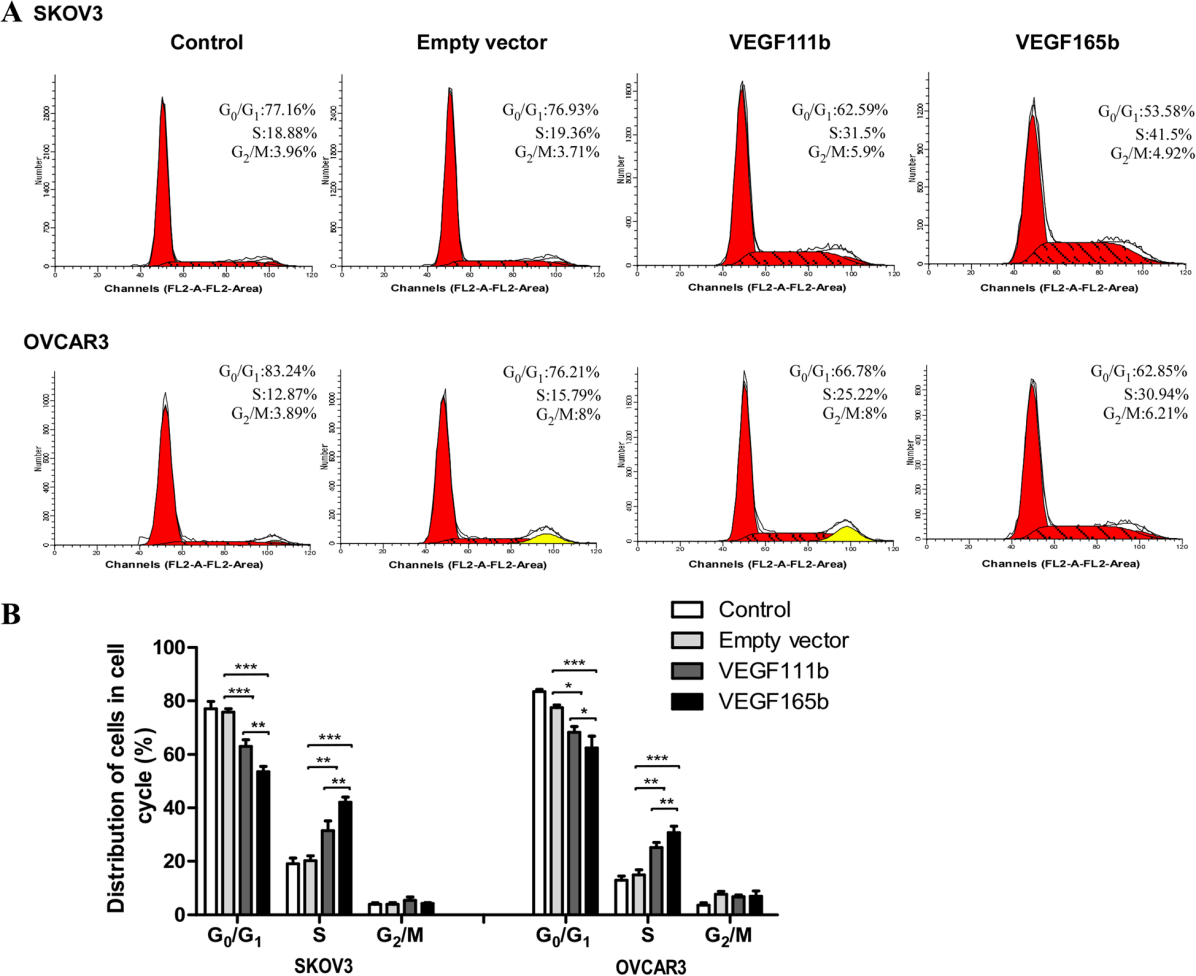
VEGF111b overexpression induces cell cycle arrest. (**a**) Detection of the proportion of ovarian cancer cells in the cell cycle stages after VEGF111b and VEGF165b is overexpressed, respectively. (**b**) The proportion of cells in the G_0_-G_1_ phase of the cell cycle decreased and the proportion of cells in the S phase increased in VEGF111b and VEGF165 group (*p < 0.05; **p < 0.01; ***p < 0.001). Bar heights represent average of data and bars represent the SEM of data obtained in three independent experiments

We then carried out cell-cycle analysis to further characterize VEGF111b inhibition effects. SKOV3 cells populations in the G_0_-G_1_ and S phases were 62.59 and 31.5 % in VEGF111b group, while 76.93 and 19.36 % in empty vector group, and 77.16 and 18.88 % in control group. Besides, OVCAR3 cells populations in the G_0_-G_1_ and S phases were 66.78 and 25.22 % in VEGF111b group, while 76.21 and 15.79 % in empty vector group, and 83.24 and 12.87 % in cortrol group (Fig. 3a). After three independent experiments, in VEGF111b group, the SKOVS cells of G_0_-G_1_ phase decreased (vs empty vector, P < 0.001; Fig. 3b) and the SKOV3 cells of S phases significantly increased (vs empty vector, *P* < 0.01; Fig. 3b). As well, the OVCAR3 cells of G_0_-G_1_ phase decreased (vs empty vector, *P* < 0.05; Fig. 3b) and the OVCAR3 cells of S phases increased in VEGF111b group (vs empty vector, *P* < 0.01; Fig. 3b). These results suggested that VEGF111b overexpression significantly induced S phase arrest compared with empty vector group and control group in ovarian cancer cells.

### VEGF111b inhibits ovarian cancer growth through inhibiting VEGF-R2/PI3K/Akt and VEGF-R2/ERK1/2 signal pathways

**Fig. 4:**
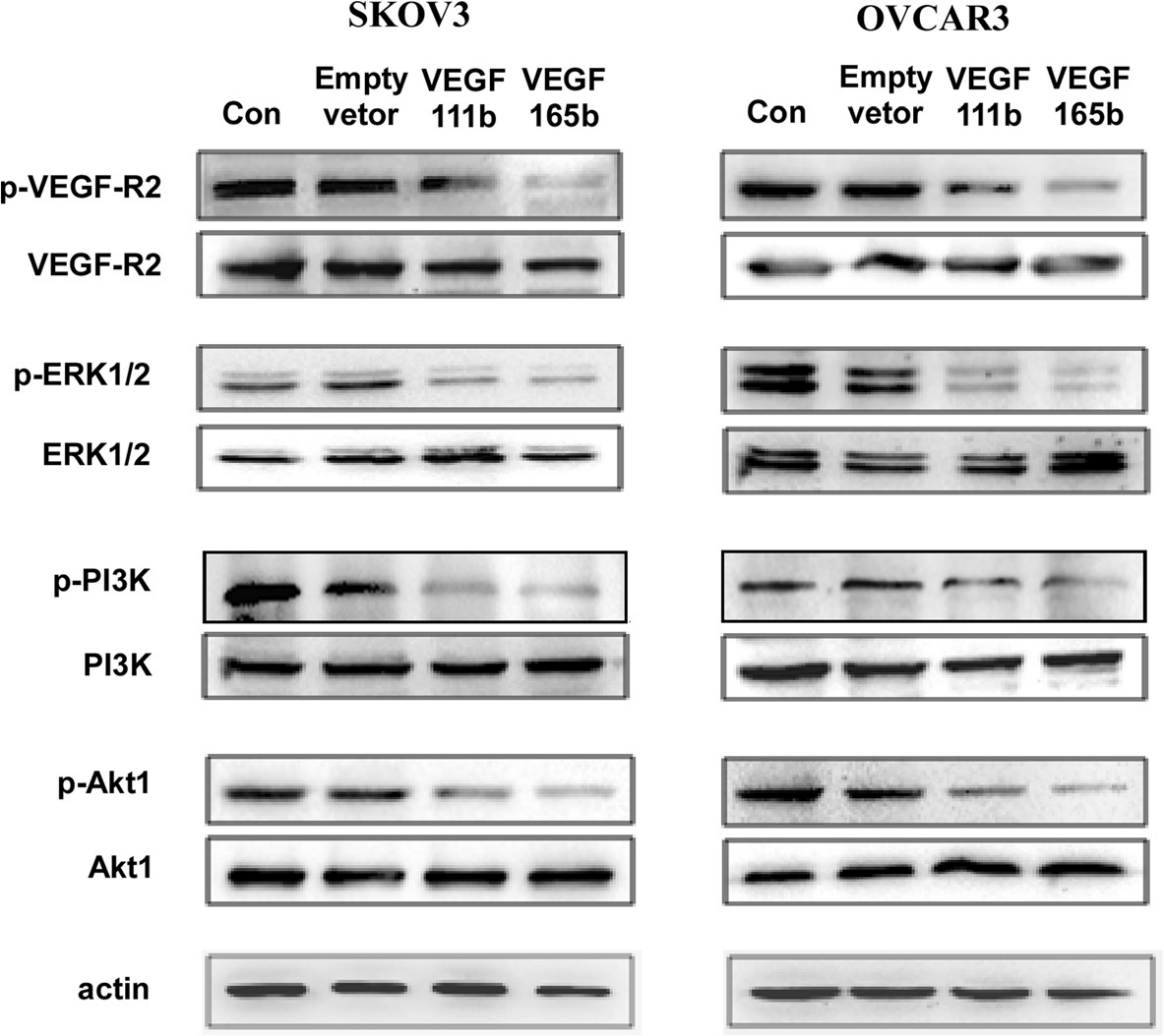
VEGF111b overexpression reduces VEGF-R2 phosphorylation and its downstream signaling. Total and phosphorylated VEGF-R2 were measured by western blot. Downstream signaling proteins of p- PI3K, p-Akt, and p-ERK1/2 were reduced by VEGF111b and VEGF165b overexpression, correspondingly. Actin served as loading control

To determine whether VEGF111b signaling on ovarian cancer cells was mediated through VEGF-R2, firstly, we investigated the VEGF-R2 protein in SKOV3 and OVCAR3 cells by western blot. We found SKOV3 and OVCAR3 cells both had VEGF-R2 protein expression. Then, we detected the levels of total and phosphorylated VEGF-R2 (p-VEGF-R2) in SKOV3 and OVCAR3 cells after which was transfected with empty lentivirus, VEGF111b and VEGF165b lentivirus and allowed to grow for 48 h. Total protein levels of VEGF-R2 were not significantly changed. By contrast, p-VEGF-R2 (Y1175) were decreased after transfection, and the phosphorylation level of the VEGF-R2 induced by VEGF165b overexpression was lower than the that induced by VEGF111b (Fig. 4). A detailed analysis of downstream signaling pathways was performed to detect the levels of phosphorylated PI3K, Akt, and p44/42 MAPK (ERK1/2). Expressions of p-PI3K, p-Akt, and p-ERK1/2 were also inhibited after VEGF111b and VEGF165b overexpression in both SKOV3 and OVCAR3 cells. Moreover, phosphorylation level of p-PI3K, p-Akt, and p-ERK1/2 induced by VEGF165b overexpression was lower than the activation induced by VEGF111b (Fig. 4). VEGF111b polyclonal antibody was added to combine VEGF111b produced by transfection. Then the phosphorylation level of p-PI3K, p-Akt, and p-ERK1/2 was restored (Additional file: 1 Figure S2).

### VEGF111b overexpression inhibits ovarian cancer growth in xenograft model

**Fig. 5:**
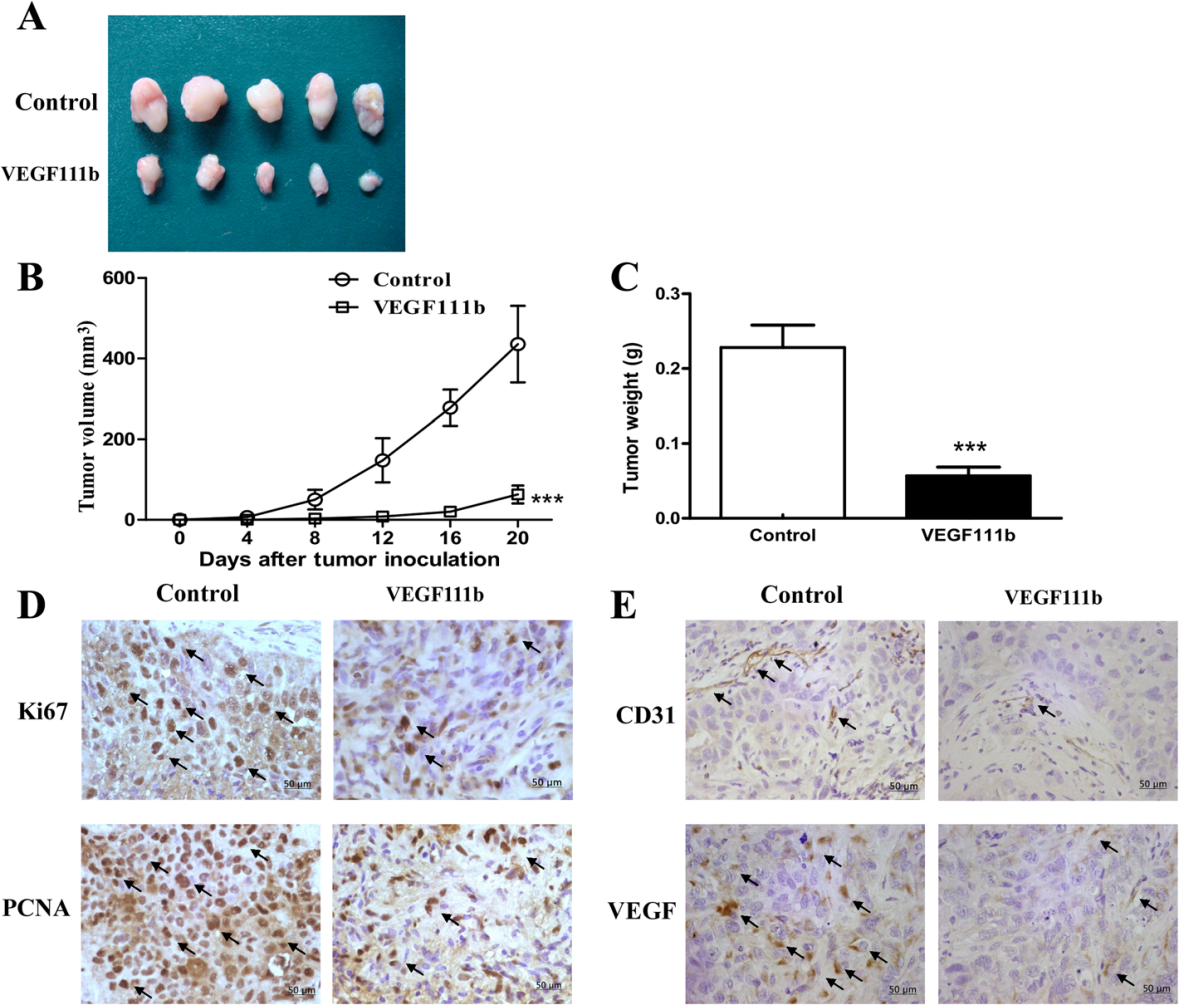
VEGF111b overexpression inhibited growth of SKOV3 tumor xenografts. After transfection with empty lentivirus and VEGF111b lentivirus, SKOV3 cells were injected subcutaneously into the upper right flank region of nude mice. Tumor volume was calculated every 4 days for 20 days. (**a**) Tumor size of the 20th day tumor incubation was displayed. (**b**) Tumor volume was measured with a caliper rule every 4 days. Datas were presented as the mean tumor volumes of mice in both VEGF111b and empty lentivirus vector groups on the days incubation (***p < 0.001). (**c**) Average tumor weight was shown at the end of the experiments (***p < 0.001). (**d**) Tumor sections were stained positive for Ki67 and PCNA. The positive products localized in nucleus (magnification, 200×). (**e**) Tumor sections were stained positive for CD31 and VEGF. The positive products localized in cytoplasm (magnification, 200×). Bar heights represent average of data and bars represent the SEM of data. *P* value <0.05 was considered statisticall

To evaluate the role of VEGF111b overexpression on tumor growth *in vivo*, we examined the effect in aectopic model of SKOV3 cells which were transfected with VEGF111b lentivirus. Tumor size on the 20th day tumor incubation was displayed (Fig. 5a). VEGF111b inhibited the growth of the SKOV3 xenografts, compared with empty vector group (Fig. 5b). The weight of excised tumors from vehicle mice was displayed (*P* < 0.001, Fig. 5c), which both suggested significant inhibition effect of VEGF111b overexpression *in vivo*. In VEGF111b xenograft model, significant decreases of cell proliferation were observed by Ki67 and PCNA (Fig. 5d). Furthermore, VEGF111b overexpression in SKOV3 xenografts led to a significant reduction in CD31 and VEGF compared with empty vector group (Fig. 5e). Reduction of CD31-marked microvessel density (MVD) in tumor xenograft samples indicated a lower angiogenesis in comparison to empty vector group. And VEGF111b significantly inhibited the expression of VEGF in SKOV3 tumors.

## Discussion

Ovarian cancer presents at advanced stage in around 75 % of women. Despite improvements in treatments such as cytotoxic chemotherapy, ovarian cancer still sets in stone in the 5-year [17]. This has led to a search for novel approaches for molecular targeted therapy. VEGF-mediated angiogenesis is a requirement in normal ovarian function, controlling the cyclical growth of ovarian follicles and maintenance of the corpus luteum [18]. Antiangiogenesis therapy has attracted more and more attention, and becomes a more general treatment target. Previously, we first presented existence of VEGF111b as a different VEGFxxxb family member, and that its mRNA and protein expression were induced in the human ovarian cancer cells by genotoxic agents such as mitomycin C [7]. Here, we detect its expression in other types of ovarian cancer cells – OVCAR3 cells, and further confirm that the expression of VEGF111b depend upon the treatment of mitomycin C inducing double-strand breaks, which is similar to VEGF111 [16]. Mitomycin C triggers double-strand breaks which is the dependent of VEGF111 expression.

The most studied VEGFxxxb family member - VEGF165b - has been clearly shown to be anti-angiogenic. Other researchers have shown that the six amino acids of exon 8b are required for the anti-angiogenic activity of VEGF165b because artificial generation of VEGF159 lacks both exons 8b, which is not angiogenic [14]. Consistent with VEGF165b, we have found that VEGF111b could also inhibit angiogenesis in previous study [7]. In the present study, our studies showed VEGF111b could perform anti-tumor effects *in vitro* and *in vivo*. VEGF111b inhibits proliferation of the ovarian cancer cells, and *in vivo* VEGF111b overexpression inhibits ovarian cancer growth in xenograft mice. Cell cycle analysis was further used to elucidate the mechanism involved in the anti-tumor effect of VEGF111b overexpression. Further, we observed the expression of VEGF-R2 in SKOV3 and OVCAR3 cells that was consistent with Ptaszynska [19]. In this study we have found that VEGF111b itself reduced the VEGF-R2 tyrosine phosphorylation and accordingly downstream signaling, the phosphorylation of PI3K, Akt, and ERK1/2. Research has showed that VEGF-R2 tyrosine kinase inhibitors can reduce the phosphorylation of PI3K, Akt, and ERK1/2 [20]. In supplementary experiments, we added VEGF111b polyclonal antibody we prepared in the conditioned medium of SKOV3 cells overexpressing VEGF111b. Then we observed the recovery of the phosphorylation levels of the VEGF-R2 and its downstream signaling pathways. It demonstrated that VEGF111b might bind to conventional VEGF-R2 with the results of reducing phosphorylation of PI3K, Akt, and ERK1/2 to play anti-tumor effects. Exon 8a has been now identified to be essential for neuropilin-1 (NRP-1) and heparin binding [12, 21]. The VEGF111b isoform that lacks this moiety are unable to bind NRP-1, resulting in reduced VEGF-R2 tyrosine phosphorylation and downstream signaling [6, 22]. In agreement with previous observations, VEGF111b inhibits ovarian cancer growth, but it is slightly less efficient than VEGF165b. This may be related to the lack of exons 5–7 in VEGF111b which affects the location of the receptor-binding interfaces in the dimeric molecule.

Mechanistically, we found that VEGF111b inhibited VEGF-R2-mediated signaling pathways, including ERK1/2 and PI3K-Akt signaling pathways. VEGF-Rs contain three subtypes: VEGF-R1, VEGF-R2, and VEGF-R3 [23]. VEGF-R2 is the major receptor, which mediates angiogenic activity of VEGF via diverse signaling pathways, including MAPK family and PI3K-Akt axis that regulate proliferation, migration, and tube formation of cells [24]. VEGF activates three MAPKs, namely ERK1/2, JNK1/2, and p38 [25]. The ERK activation results in an increased proliferation of cells [26]. In addition, the activation of PI3K-Akt axis has been shown to promote cell survival, migration, and cytoskeletal rearrangement [27]. Here, we demonstrated that VEGF111b inhibits ovarian cancer growth through inhibiting VEGF-R2 and downstream ERK1/2 and PI3K-Akt signaling pathways. Similar to VEGF111 [16], we cannot detect VEGF111b mRNA and protein in human ovarian cancer cells OVCAR3 and SKOV3 without mitomycin C treatment. The expression of VEGF111 and VEGF111b isoforms in human ovarian tumors with and without treatments needs to be investigated in future clinical study.

In conclusion, our results revealed that VEGF111b has an inhibitory effect on ovarian cancer cells through inhibition of VEGF-R2 tyrosine phosphorylation and downstream signaling pathways, and this will potentially open a new possible avenue for treating ovarian cancer.

## Additional file

Additional file 1: **Supplementary data.**
