# Supplementary material for: VEGF111b, a C-terminal splice variant of VEGF-A and induced by mitomycin C, inhibits ovarian cancer growth
Source: J Transl Med. 2015 May 20;13:164. doi: 10.1186/s12967-015-0522-0 (PMC4480579; doi:10.1186/s12967-015-0522-0)

**Supplementary Data**

**Figure S1.** (A) Production of polyclonal antibody VEGF111b. Synthetic peptide fragments of the 8 amino acids CRSLTRKD in the C-terminal sequence of VEGF111b were coupled to KLH and used to immunize two male New Zealand long ear rabbits. After the immunotherapy, ear vein blood was taken for ELISA analysis. When the titers reached to 1.28×10^5^ and met the requirements of the experiment, serum was collected to purify VEGF111b polyclonal antibody by ammonium sulfate precipitation. (B) After SKOV3 and OVCAR3 cells were infected with VEGF111b and VEGF165b lentivirus respectively, western blot showed that our homemade VEGF111b polyclonal antibody could specifically recognize VEGF111b protein, but does not recognize the VEGF165b protein. (C) We used our homemade VEGF111b antibody to combine different MOI transfection of VEGF111b, VEGF111 and VEGF165b, and different concentrations of VEGF165. According to the antibody affinity testing for different VEGF family members, we found that the homemade VEGF111b antibody has highest affinity to VEGF111b protein.

**Figure S2.** In our study VEGF111b itself inhibited the phosphorylation of VEGF-R2 and its downstream signaling pathways. Research has showed that VEGF-R2 tyrosine kinase inhibitors can reduced the phosphorylation of PI3K, Akt, and ERK1/2 (Ref 19). Therefore, we added the polyclonal antibody of VEGF111b we prepared in the conditioned medium of SKOV3 cells overexpressing VEGF111b. Then VEGF111b polyclonal antibody restored the phosphorylation of VEGF-R2, PI3K, Akt, and ERK1/2. VEGF111b inhibited the expressions of p-PI3K, p-Akt, and p-ERK1/2, but VEGF111b pAb restored the phosphorylation of VEGF-R2, PI3K, Akt, and ERK1/2 (line 4).

Supplementary Figure S1


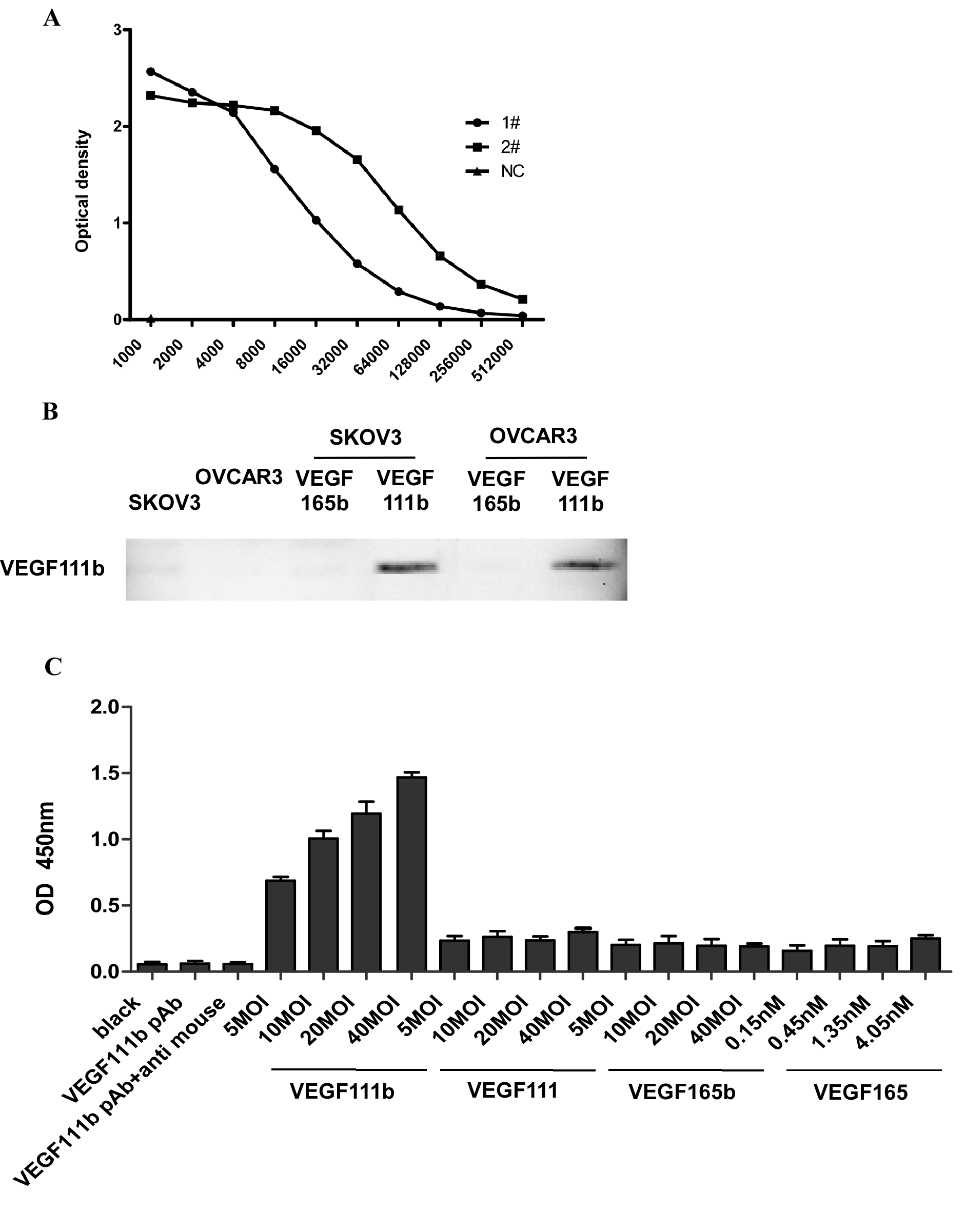


Supplementary Figure S2


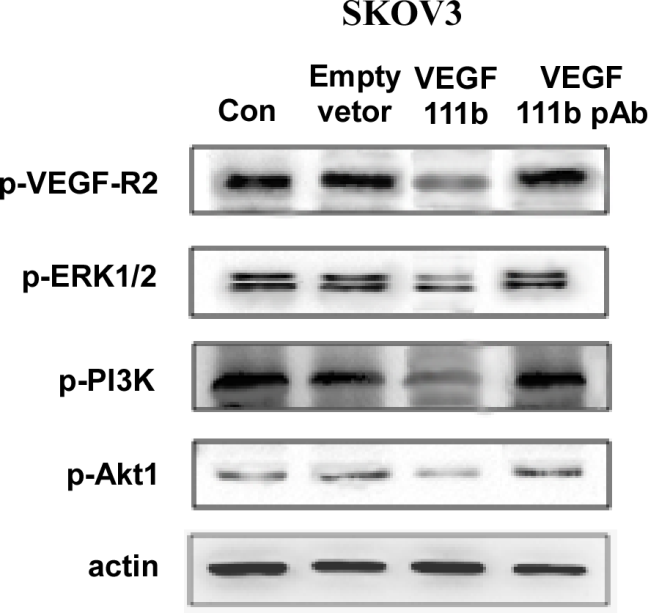

Supplement: Supplementary file 1 — Supplementary data. [file 12967_2015_522_MOESM1_ESM.docx]
